# Low‐dose computed tomography lung cancer screening: Clinical evidence and implementation research

**DOI:** 10.1111/joim.13480

**Published:** 2022-03-24

**Authors:** Harriet L. Lancaster, Marjolein A. Heuvelmans, Matthijs Oudkerk

**Affiliations:** ^1^ Department of Epidemiology University Medical Center Groningen University of Groningen Groningen The Netherlands; ^2^ Institute for Diagnostic Accuracy Groningen The Netherlands; ^3^ Faculty of Medical Sciences University of Groningen Groningen The Netherlands

**Keywords:** early detection, LDCT, lung cancer, pulmonary nodules, screening

## Abstract

Lung cancer causes more deaths than breast, cervical, and colorectal cancer combined. Nevertheless, population‐based lung cancer screening is still not considered standard practice in most countries worldwide. Early lung cancer detection leads to better survival outcomes: patients diagnosed with stage 1A lung cancer have a >75% 5‐year survival rate, compared to <5% at stage 4. Low‐dose computed tomography (LDCT) thorax imaging for the secondary prevention of lung cancer has been studied at length, and has been shown to significantly reduce lung cancer mortality in high‐risk populations. The US National Lung Screening Trial reported a 20% overall reduction in lung cancer mortality when comparing LDCT to chest X‐ray, and the Nederlands‐Leuvens Longkanker Screenings Onderzoek (NELSON) trial more recently reported a 24% reduction when comparing LDCT to no screening. Hence, the focus has now shifted to implementation research. Consequently, the 4‐IN‐THE‐LUNG‐RUN consortium based in five European countries, has set up a large‐scale multicenter implementation trial. Successful implementation of and accessibility to LDCT lung cancer screening are dependent on many factors, not limited to population selection, recruitment strategy, computed tomography screening frequency, lung‐nodule management, participant compliance, and cost effectiveness. This review provides an overview of current evidence for LDCT lung cancer screening, and draws attention to major factors that need to be addressed to successfully implement standardized, effective, and accessible screening throughout Europe. Evidence shows that through the appropriate use of risk‐prediction models and a more personalized approach to screening, efficacy could be improved. Furthermore, extending the screening interval for low‐risk individuals to reduce costs and associated harms is a possibility, and through the use of volumetric‐based measurement and follow‐up, false positive results can be greatly reduced. Finally, smoking cessation programs could be a valuable addition to screening programs and artificial intelligence could offer a solution to the added workload pressures radiologists are facing.

## Graphical abstract

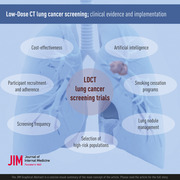

## Introduction

Lung cancer led to 1.8 million deaths in 2020, and remains the leading cause of cancer‐related deaths globally [1]. Notable therapeutic improvements, such as the introduction of immunotherapy, have led to improved outcomes in a limited number of patients with late‐stage (stage IV) lung cancer; however, prognosis remains poor for the majority of lung cancer patients [2]. Low‐dose computed tomography (LDCT) lung cancer screening (LCS) offers a promising solution to the early detection of lung cancer, and subsequently could significantly reduce lung cancer mortality. Multiple LCS studies have provided indisputable evidence for the benefits of LDCT LCS [3, 4]. However, LDCT LCS programs are yet to be implemented on a large scale globally. This review will highlight both the benefits and future challenges associated with the large‐scale implementation of LCS programs. We will examine the existing supporting evidence for LDCT LCS, gathered during extensive national LCS trials. Additionally, we will outline important obstacles that still need to be overcome, such as optimal selection of screening population, screening interval, the most appropriate nodule management protocol, cost effectiveness, infrastructure, participant compliance, and incorporation of smoking cessation programs. All of the aforementioned are crucial factors in the successful global implementation of LDCT LCS.

## LDCT LCS trial evidence

Various randomized control trials (RCTs) have now taken place in both the United States and Europe to evaluate the effectiveness of LDCT LCS, many of which show promising results.

The National Lung Screening Trial (NLST) and the Nederlands‐Leuvens Longkanker Screenings Onderzoek (NELSON) trial are two trials with adequate power to evaluate reduction in lung cancer mortality, both of which showed LDCT screening could significantly reduce lung cancer mortality in a high‐risk population [3, 5, 6]. The NLST began in 2002 and recruited 53,454 participants, aged 55–74 years, who were either current or former smokers with at least 30‐pack‐years smoking history and who were at high risk of developing lung cancer. Participants were randomized into either a LDCT or chest X‐ray screening group and underwent annual screening over 3 years. This trial reported an overall 20% (95% confidence interval [CI], 6.8–26.7; *p* = 0.004) reduction in lung cancer mortality after 6.5‐years follow‐up when using LDCT compared to chest X‐ray for LCS [3]. In contrast, the NELSON trial compared LDCT screening at baseline, year 1, year 3, and year 5.5 to no screening. This study, which began its first recruitment in 2003, included 15,792 participants aged 50–74 years with a high risk of developing lung cancer—both smokers and former smokers with ≥30 pack‐years smoking history. In 2020, the NELSON trial published their final results, reporting a cumulative rate ratio for lung cancer death of 0.76 (95% CI, 0.61–0.94; *p* = 0.01) in the screening arm relative to the control arm after a follow‐up of 10 years [4].

Additional RCTs that have taken place in Europe, despite being underpowered for the evaluation of lung cancer mortality reduction, have shown similar encouraging results. The Multicentric Italian Lung Detection (MILD) trial compared LDCT screening to no intervention in participants aged ≥49 years with ≥20 pack‐years smoking history, and found a significant 39% reduction in the cumulative risk of lung cancer mortality at 10 years in the LDCT arm of the trial (hazard ratio [HR] 0.61; 95% CI, 0.30–0.95; *p* = 0.02) [7]. The German Lung Cancer Screening Intervention (LUSI) RCT also compared LDCT to no intervention, and found a statistically significant reduction in lung cancer mortality in women (HR 0.31; 95% CI, 0.10–0.96; *p* = 0.04) in the LDCT arm [8]. Numerous other trials also showed a nonsignificant reduction in lung cancer mortality when comparing LDCT screening to either chest X‐ray or no intervention [9, 10, 11, 12, 13]. An overview of the aforementioned RCTs, inclusive of total participant number, inclusion criteria, and outcomes, can be seen in Table 1.

**Table 1:** Overview of LDCT lung cancer screening RCT trials; inclusive of selection criteria and main findings

Study	Comparison	N	Selection criteria	Main findings
Powered RCTs				
NLST (3)	LDCT versus CXR	53,454	Age 55–75 years, ≥30‐pack‐years smoking history, former smokers quit <10 years previous	LDCT lead to a 20% (95% CI, 6.8–26.7; *p* = 0.004) reduction in lung cancer mortality after 6.5‐year follow‐up
NELSON (4)	LDCT versus no intervention	15,792	Age 50–74 years, ≥15 cigarettes/day for 25 years or ≥10 for over 30 years, former smokers quit <10 years previous	LDCT lead to a 24% (HR 0.76; 95% CI, 0.61–0.94; *p* = 0.01) reduction in lung cancer mortality at 10‐year follow‐up
Underpowered RCTs				
MILD (7)	LDCT versus no intervention	4099	Age ≥49 years, ≥20 pack‐years smoking history, former smokers quit ≤15 years previous	LDCT lead to a 39% (HR 0.61; 95% CI, 0.30–0.95; *p* = 0.02) reduction in lung cancer mortality at 10‐year follow‐up
LUSI (8)	LDCT versus no intervention	4052	Age 50–69 years, ≥15 pack‐years smoking history, former smokers quit <10 years previous	LDCT lead to a reduction in lung cancer mortality in women only (HR 0.31; 95% CI, 0.10–0.96; *p* = 0.04)
DANTE (9)	LDCT versus no intervention	2811	Age 60–74 years, ≥20 pack‐years smoking history, former smokers quit <10 years previous	No reduction in lung cancer mortality was found (HR.0.993; 95% CI, 0.688–1.433)
DEPISCAN (10)	LDCT versus CXR	765	Age 50–75 years, ≥15 pack‐years smoking history, former smokers quit <15 years previous	LDCT lead to the detection of eight lung cancers versus one when using CXR
DLCST (11)	LDCT versus CXR	4104	Age 50–70 years, ≥20 pack‐years smoking history, former smokers quit <10 years previous	No reduction in lung cancer mortality was found (HR 1.03; 95% CI, 0.66–1.6; *p* = 0.888)
ITALUNG (12)	LDCT versus no intervention	3206	Age 55–69 years, ≥20 pack‐years smoking history, former smokers quit <10 years previous	Nonsignificant reduction in lung cancer mortality in LDCT arm (HR 0.70; 95% CI, 0.47–1.03; *p* = 0.07)
UKLS (13)	LDCT versus no intervention	4055	Age 50–75 years, LLPv2 5‐year lung cancer risk score ≥5%	Nonsignificant reduction in lung cancer mortality in LDCT arm (HR 0.65; 95% CI, 0.41–1.02; *p* = 0.062)

Abbreviations: CI, confidence interval; CXR, chest X‐ray; DANTE, detection and screening of early lung cancer with Novel imaging TEchnology; DLCST, Danish Lung Cancer Screening Trial; HR, hazard ratio; LDCT, low‐dose computed tomography; LLP, Liverpool Lung Project risk model; LUSI, Lung Cancer Screening Intervention; MILD, Multicentric Italian Lung Detection; N, number of participants; NELSON, Nederlands‐Leuvens Longkanker Screenings Onderzoek; NLST, National Lung Screening Trial; RCT, randomized control trial; UKLS, UK Lung Cancer Screening.

The US Preventive Services Task Force, as an independent, volunteer panel of national experts in disease prevention and evidence‐based medicine, graded the strength of evidence as moderate before the final publication of the NELSON data because of the unknown consistency of just one single powered study. After including the NELSON results, the evidence was graded as high and the recommendation followed for annual LCS with LDCT in adults aged 50–80 years who have a 20 pack‐year smoking history and currently smoke or have quit within the past 15 years. Screening should be discontinued once a person develops a health problem substantially limiting life expectancy or the ability or willingness to have curative lung surgery [14].

## Optimal selection of a screening population

**Fig. 1:**
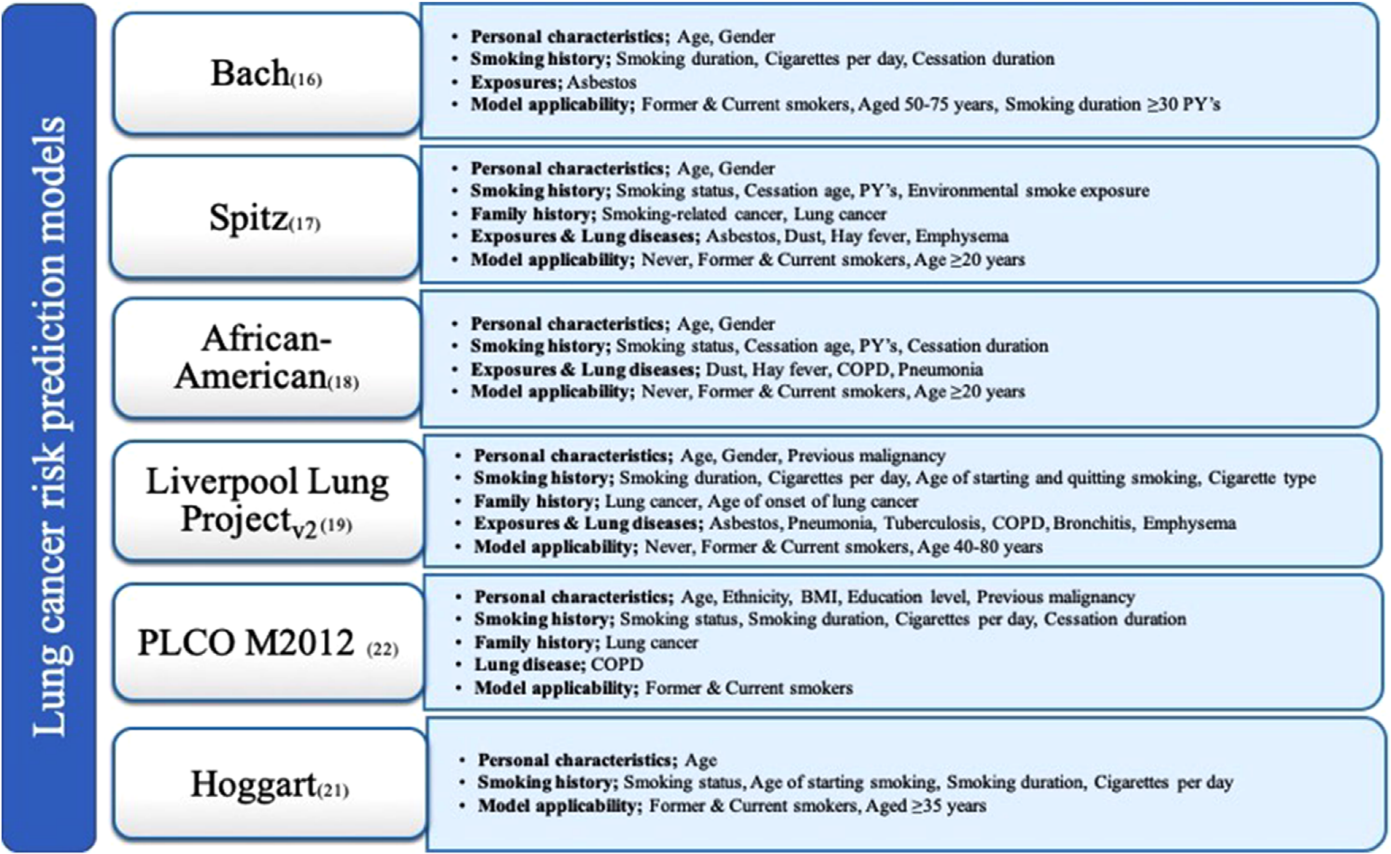
Summary of variables included in existing lung cancer risk‐prediction models. BMI, body mass index; COPD, chronic obstructive pulmonary disease; PY, pack‐years.

Appropriate selection of a high‐risk population for LDCT LCS is imperative for screening to be effective and to reduce associated harms, such as radiation exposure. Most LDCT LCS trials have selected participants based on age and smoking status, and as mentioned previously, they have shown significant reduction in lung cancer mortality when using LDCT screening. However, age and smoking status are not the only lung cancer risk factors. Family history, genetic polymorphisms, existing respiratory illnesses, ionizing radiation, occupational exposures, and air pollution have all been linked to an increased risk of lung cancer [15]. Consequently, multiple lung cancer risk‐prediction models have been developed and externally validated [16, 17, 18, 19, 20, 21, 22]. So far, the UK Lung Cancer Screening (UKLS) trial is the only RCT to use a lung cancer risk‐prediction model to select a high‐risk population for an LCS trial [23]. The UKLS trial included participants aged 50–75 years, with ≥5% risk of developing lung cancer within 5 years based on the LLP_v_2 risk model. This risk model was externally validated on data from three independent studies and showed modest to good discrimination, with an area under the curve (AUC) of 0.67–0.82. The following risk factors are included in the model to determine a 5‐year risk of lung cancer: age, sex, history of malignancy, smoking duration, family history of lung cancer including age of onset, asbestos exposure, and history of pneumonia [24]. The PLCO_M2012_ risk model has also shown promising results in calculating a 6‐year risk of lung cancer. After external validation, this model shows good discrimination with an AUC of 0.797. When compared to the NLST criteria for selecting a high‐risk population, the PLCO_M2012_ risk model had both a higher sensitivity (83.0% vs. 71.1%; *p* < 0.001) and positive predictive value (4.0% vs. 3.4%, *p* = 0.01), and no loss of sensitivity [22]. Therefore, the use of a risk‐prediction model for participant selection in an LCS program could improve effectivity and research should now be focused on further fine tuning and independent validation of existing risk‐prediction models. An overview of the variables included in the various lung cancer risk‐prediction models can be seen in Fig. 1.

## LDCT‐screening frequency

LDCT LCS, as with any screening program, does not come without possible harm, one of which is radiation exposure that participants receive during their scan. Therefore, it is important to consider factors that affect the benefit–harm ratio of LCS, one of which is screening interval. A short screening interval could lead to a reduction in interval cancers (cancers detected between screening rounds), and in turn also a reduction in the detection of late‐stage lung cancer. However, a short interval does involve increased radiation exposure, costs, and a possible increase in false‐positive results, hence the importance of finding the appropriate balance.

The NLST had annual screening over a period of 3 years [25], whereas the NELSON trial used screening intervals of 1 year, then 2 years, and then 2.5 years. After an interval of 2.5 years, more interval cancers were detected, of which significantly more were late stage. Consequently, it was concluded that a screening interval of 2.5 years likely leads to a decrease in effectivity of a screening trial [26]. However, the use of annual versus biennial screening is still being debated. The MILD trial compared annual versus biennial screening, and found no significant difference in the number of interval cancers [27].

Based on existing evidence from LCS trials and modeling studies from the Cancer Intervention and Surveillance Modeling Network (CISNET) [28], Canada and the United States recommend annual screening intervals [28, 29]. However, in favor of conserving costs, other countries may choose biennial screening. Baldwin et al. suggest several approaches for selecting screening intervals [30]. The first is adjusting screening frequency based on a person's lung cancer risk, meaning those with a higher 5‐year risk of lung cancer would have a shorter screening interval. The second is adjusting screening frequency based on the presence of baseline lung nodules and new nodules detected. However, as the others state, this is not supported by evidence from existing LCS trials.

## Lung‐nodule management

For LCS to be effective, lung‐nodule management needs to be optimal. Based on existing LDCT‐screening trial data, approximately 50% of the screened participants have ≥ 1 nodule detected at baseline [2, 31, 32]. Importantly, more than half of the nodules detected are small in size—<50 mm^3^ or maximum diameter <5 mm [33, 34, 35]—and lung cancer probability does not correlate with the number of nodules detected [36]. Furthermore, in the NELSON trial, 5%–7% of participants who underwent LDCT screening had a new solid nodule at follow‐up. Even when small in size, these new nodules had a higher risk of malignancy; however, the number of new nodules did not directly relate to malignancy risk [37, 38]. These results suggest that each lung nodule should be assessed on an independent basis, for which multiple lung‐nodule management guidelines have been introduced.

Lung nodules are assessed predominantely based on size, growth, and type. During the NLST, lung‐nodule size measurement was based on maximum diameter. This was also recommended in the early version (1.0) of the Lung Imaging Reporting and Data System (Lung‐RADS) guideline and the Fleischner Society guidelines [39, 40]. However, the NLST reported a substantial number of false‐positive results (24%), which is thought to be largely due to the use of maximum diameter measurements. This hypothesis was supported when the NELSON trial reported a reduction in false‐positive results through the use of volumetric size measurements [4, 41]. Henceforth, volume‐based lung‐nodule measurement has been recommended in/added to subsequent guidelines—Lung‐RADS v1.1, European Position Statement on Lung Cancer (EUPS), British Thoracic Society guidelines, and NELSON‐Plus protocol [2, 42, 43, 44]. Nodule growth at follow‐up screening can also be more accurately detected when using volumetric measurements in place of diameter, and can be used for the calculation of volume doubling time (VDT) [45]. VDT represents the exponential growth of a lung nodule, and can subsequently be used for determining nodule management and follow‐up.

**Fig. 2:**
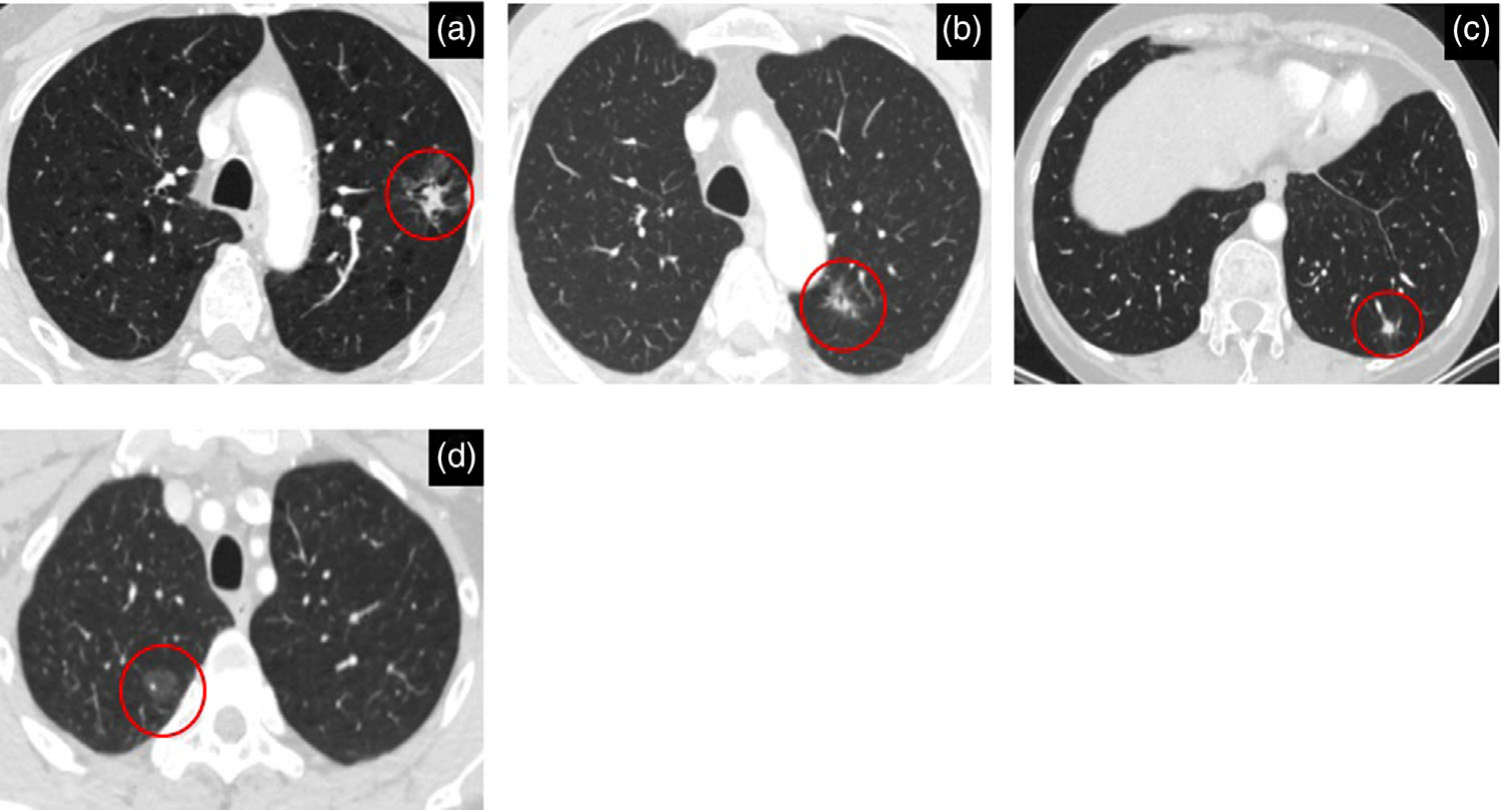
Computed tomography images showing solid and subsolid lung nodules: (a) mixed ground‐glass malignant (invasive adenocarcinoma) nodule peripherally situated in the left upper lobe measuring 25 mm max. diameter. (b) Mixed ground‐glass malignant (invasive adenocarcinoma) nodule situated peripherally in the left upper lobe measuring 14 mm max. diameter. (c) Solid benign nodule is situated peripherally in the left lower lobe measuring 248 mm^3^. (d) Pure ground‐glass malignant (adenocarcinoma in situ) nodule situated peripherally in the right upper lobe measuring 14 mm max. diameter.

The nodule type has also been shown to be consequential to the risk of malignancy. Nodules can be classified into calcified and noncalcified, with the latter being further classified into solid and subsolid (part‐solid and pure‐ground glass). Solid nodules are most prevalent in lung cancer screening. However, subsolid nodules are associated with a higher malignancy risk, albeit they are usually detected at a premalignant or early stage [46, 47, 48]. Furthermore, a nodule's risk of malignancy can also be related to other distinguishing characteristics, such as location and attachment. In the NELSON trial for example, 82.2% of adenocarcinomas were detected in the periphery (outer one‐third of the hilar‐costal diameter) of the lungs and were attached to the pleura compared to 17.8% detected in the middle or centrally (inner two‐thirds of the hilar‐costal diameter). Additionally, 45.0% of all lung cancers were situated in the right upper lobe [49]. Thus, these results affirm the suggestion that nodule management should be decided on an independent nodule to nodule basis. Computed tomography (CT) images of solid and subsolid nodules can be seen in Fig. 2.

## Cost effectiveness of LDCT LCS

Numerous LCS cost‐effectiveness studies have been performed and results have been variable. A health technology assessment performed by Snowsill et al. in 2018 evaluated the use of LDCT in screening for lung cancer in high‐risk UK populations and concluded that the cost effectiveness and clinical effectiveness remained uncertain [50]. A more recently published microsimulation modelling study by Du et al. in 2020 based on a Dutch population found LDCT LCS to be cost effective in a high‐risk population. They reported LDCT LCS was most cost effective when performed annually in males aged 55–80 years who were heavy smokers, and biennially in females aged 50–80 years who were heavy smokers [51]. Similar results were found in a cost‐effectiveness analysis performed in Switzerland [52], and to a lesser degree in a German population [53].

Outside of Europe, similar analyses have been performed. In the United States, Criss et al. used four independently developed microsimulation models to evaluate the cost effectiveness of LCS according to the maximum age recommendations of the US Preventative Services Task Force (USPSTF) (80 years), Centers for Medicare and Medicaid Services (CMS) (77 years), and the NLST (74 years). They reported all of the aforementioned screening strategies to be cost effective, with incremental cost‐effectiveness ratios (ICERs) of $96,700, $68,600, and $49,200, respectively—all of which fall under the willingness‐to‐pay threshold in the United States of $100,000 per quality‐adjusted‐life‐year (QALY) [54]. In Canada, Ten Haaf et al. found LDCT screening of persons aged 55–75 with a smoking history of ≥40 pack years resulted in a cost‐effectiveness ratio of $41,136 Canadian dollars per life‐years gained, again falling under the threshold of $50,000 Canadian dollars [55], whereas a simulation study in an Australian population based on NLST selection criteria found LDCT screening unlikely to be cost effective. They reported an ICER of $233,000 Australian dollars per QALY gained, falling above the willingness‐to‐pay threshold in Australia of $30,000–$50,000 Australian dollars per QALY [56].

The cost effectiveness of LDCT LCS undoubtedly varies according to the country in which screening is to be implemented. This suggests that the implementation strategy of the LCS program should be specific to the country in which it is to be implemented. Cost‐effectiveness analyses should therefore now be focused on country‐specific health and social care infrastructure and perspectives.

## Artificial intelligence in LCS

Implementing LDCT LCS globally is no easy feat, essentially due to the substantial increase in workload that radiologists face. Radiologists are under ever‐increasing pressure in the clinic due to significant workforce shortages and an unrelenting demand for radiological services [57]. Demand will only increase with the implementation of CT LCS, and consequently a solution is required. Artificial intelligence (AI) could offer the solution.

### AI and lung‐nodule detection

AI systems that can be used in LCS have shown significant improvement over the decades. Different methods of using an AI system to assist a human reader have been investigated—as a first reader, second reader, or a concurrent reader [58]. AI as a first reader is the optimal strategy when looking to reduce the radiologists’ workload, as the human reader only reviews the nodules deemed clinically significant by the AI system. However, this method requires the highest degree of accuracy to avoid dangerous false‐negative (undetected clinically significant nodules) results. AI as second reader works to improve the overall performance of the human reader. The human reader performs an independent initial read, followed by an AI‐system read, and subsequently comparisons are made to identify missed or misclassified nodules. Lastly, a concurrent read involves the human reader using the entire read of the AI system to assist with their final interpretation.

Computer‐aided detection (CAD) systems that can act as a “second reader” for the radiologist have shown promise in improving the accuracy of nodule detection [59]. Rubin et al. showed that CAD used as a second reader for pulmonary nodule detection substantially increased mean sensitivity from 63% (range, 56%–67%) to 76% (range, 73%–78%) when compared to a conventional double human read [60]. Liang et al. retrospectively compared four CAD systems to radiologists’ reads performed in the I‐ELCAP study. They showed that CAD systems were able to identify up to 70% of lung cancers that were missed by radiologists, but missed 20% of lung cancers that were identified by radiologists. These results also suggest that the use of these CAD systems as a second reader would be advantageous in LDCT LCS [61]. This promising result has also been replicated in numerous other publications [62, 63, 64].

Concurrent reading using CAD systems has also been proven to be effective. Silva et al. investigated the use of a CAD system for the detection of subsolid nodules in the MILD trial. In this study, CAD had a higher sensitivity than visual readings; however, human visual confirmation of CAD markings was required to reduce the number of false‐positive findings. This outcome suggests that a concurrent reading using both CAD and human visual reading provides the optimal detection of subsolid lung nodules [65].

Despite significant supporting evidence showing the value of CAD systems in detection of pulmonary nodules, they are yet to be implemented in clinical practice. This is largely due to suboptimal sensitivity and specificity outcomes associated with the existing systems. Continuous fine tuning of existing CAD systems will hopefully lead to a reduction in false‐negative and positive results, and in turn they could be successfully implemented in LDCT LCS programs.

### AI and lung‐nodule classification

A different approach to the use of AI in LDCT LCS is lung‐nodule classification using radiomics or deep learning (DL) models to distinguish between benign and malignant nodules. Radiomics are computer algorithms that can extract a large amount of quantitative data from regions of interest on CT scans. These data can, for example, include variables relating to shape, voxel gray level intensity, and spatial relationships. Thus, the aim of radiomics in LCS is to develop new imaging biomarkers that could help differentiate between malignant and benign nodules [66]. Liu et al. used radiomic models to differentiate between adenocarcinomas and benign lesions detected using LDCT, and found a higher specificity and equivalent sensitivity when compared to the Lung‐RADS classification system [67]. Radiomics could also help with the timely planning of individual‐based LCS intervals, as previously suggested by Wang et al. This group proposed a radiomics‐based follow‐up schedule and assessed its performance in comparison to five existing management guidelines. They reported that their proposed radiomic‐based schedule performed better than the five existing guidelines when looking at timely lung cancer diagnosis and preventing unnecessary follow‐up screening [68]. Similarly, DL models show potential in the field of LCS. Heuvelmans et al. trained a lung cancer prediction convolutional neural network (LCP‐CNN) using NLST data to predict the malignancy score for lung nodules. The LCP‐CNN performance was excellent in ruling out benign lung nodules when tested independently in a European trial dataset [69]. Baldwin et al. tested the same LCP‐CNN model in a UK dataset, and compared it with the Brock University model for the estimation of lung‐nodules malignancy risk. They found that LCP‐CNN was better able to discriminate between benign and malignant nodules than the Brock model [70]. Liu et al. compared the performance of a DL model to that of radiologists. Their results showed that the performance of the DL model was not dependent on the radiation dose, patient age, or the CT scanner used, and when used by a radiologist their performance improved and overall reading time decreased [71].

Alternatively, in place of AI differentiating between benign and malignant lung nodules, workload reduction can be achieved by correctly classifying nodules by size. A recent study on the performance of AI for categorization of lung nodules based on volumetric size measurement showed that AI could outperform four experienced radiologists when looking at negative misclassifications, resulting in a possible workload reduction of up to 86.7% [72].

## Participant recruitment and adherence

The recruitment and adherence of participants is a challenge that should not be underestimated. Several factors can affect participant recruitment and adherence, such as socio‐economic status, age, gender, smoking status, and family history of lung cancer.

**Fig. 3:**
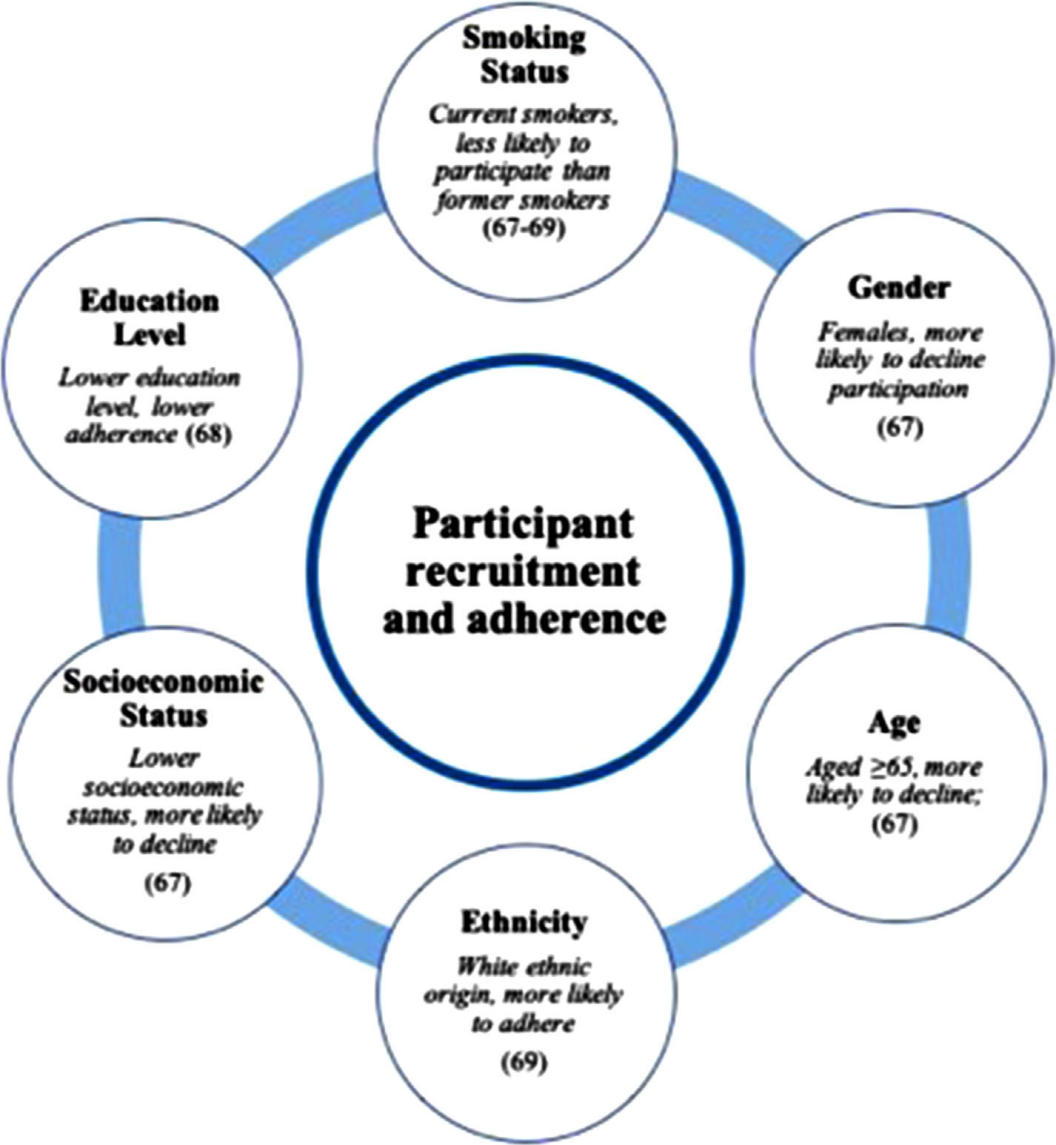
Summary of factors associated with participant recruitment and adherence in low‐dose computed tomography lung cancer screening programs.

Ali et al. analyzed barriers to participation in the UKLS trial. They found that persons who declined participation were more likely: female gender (odds ratio [OR] 0.64, *p* < 0.001), individuals >65 years of age (OR 0.73, *p* < 0.001), current smokers (OR 0.70, *p* < 0.001), of lower socioeconomic status (OR 0.56, *p* < 0.001), and had higher affective risk perception (OR 0.52, *p* < 0.001) [73]. A study by Lopez‐Olivo et al. looked at participant adherence to LCS in the United States. They found current smokers were less likely than former smokers to adhere to LCS (OR 0.70; 95% CI, 0.62–0.80), and persons who had completed ≥4 college years showed increased adherence than those who had not (OR 1.5; 95% CI, 1.1–2.1) [74]. Kim et al. analyzed adherence in participants screened for lung cancer within the Lung Population‐based Research to Optimize the Screening Process (PROSPR) Consortium. In a multivariable analysis, they found that Black participants had a lower adherence in comparison to White patients (OR 0.79; 95% CI, 0.66–0.94), and former smokers had increased adherence when compared with current smokers (OR 1.33; 95% CI, 1.19–1.49) [75]. A summary of factors associated with participant recruitment and adherence can be seen in Fig. 3.

To overcome participant recruitment and adherence issues, various techniques have been investigated. Lam et al. reported interventions, including dedicated program coordinators, reminder letters and calls, and mobile CT scanners, helped to reduce nonadherence in LDCT LCS [76]. For the successful implementation of LCS, adherence issues must be overcome. Further research into recruitment and adherence interventions would therefore be beneficial before LCS is implemented widespread.

## Incorporation of smoking cessation programs

LCS should not replace preventative measures such as smoking cessation programs. Nevertheless, lung cancer mortality cannot be reduced by preventative measures alone. Therefore, it has been recommended that smoking cessation programs be integrated into LCS. The EUPS suggested smoking cessation guidance be given to all current smokers recruited in LCS programs [43].

When association between smoking abstinence and mortality in NLST participants has been assessed, it is reported that lung cancer mortality reduction is greater when abstinence is combined with LCS [77, 78]. Ashraf et al. examined the smoking habits of Danish Lung Cancer Screening Trial (DLCST) participants and reported an increase in the annual point prevalence quit rate from 11% to 24% over the five screening rounds, with no relapse amongst ex‐smokers [79]. Similar positive effects of smoking cessations programs were also found in the ITALUNG trial. Pistelli et al. report a threefold significantly greater probability of quitting smoking when participants were enrolled in a smoking cessation program. Furthermore, smoking cessation was associated with male gender, lower pack‐years, and the presence of pulmonary nodules at baseline [80]. In UKLS participants, LCS also provided a teachable moment, with smoking cessation rates of 14% versus 8% at baseline in the screened versus control group, respectively (OR 2.38; 95% CI, 1.56–3.64, *p* < 0.001). In this trial, participants requiring additional investigations had an increased likelihood of quitting long term when compared with the control group (OR 2.29; 95% CI, 1.62–3.22, *p* = 0.007) [81]. In the NELSON trial, the screening group also reported high smoking abstinence rates (14.5%); however, higher rates were still seen in the control group [82].

LCS offers a teachable moment for smoking cessation and therefore the integration of cessation programs should be considered. Further research into the optimal strategy for such programs is still necessary, along with further behavioral research.

## Implementation pilots and studies

Multiple countries, including the UK, Croatia, and Poland, have now started pilot testing LCS programs [83, 84, 85]. However, as previously discussed, there are many factors that affect successful implementation, and implementation will require continuous monitoring to maintain optimal standards. Henceforth, the 4‐IN‐THE‐LUNG‐RUN project (an acronym for Towards Individually tailored Invitations, screening INtervals and INtegrated comorbidity reducing strategies in LCS) has been set up with the ultimate goal of implementing a Europe‐wide, cost‐effective volume‐based CT LCS program for high‐risk individuals, taking into consideration individuals’ backgrounds and gender [86]. The 4‐IN‐THE‐LUNG‐RUN project is a multicentered implementation trial, and will include participants from five European countries. It is hoped that this trial will provide answers to the remaining questions surrounding LCS implementation in Europe.

## Conclusion

LCS through the use of LDCT can reduce lung cancer mortality. This has now been undeniably proven in multiple RCTs. Therefore, LDCT LCS in high‐risk populations is on the brink of implementation. However, to achieve optimal outcomes, research into factors associated with LDCT‐screening implementation is still necessary. Shifting the focus to this type of research will help to achieve the fundamental goal of implementing accessible, affordable, and applicable CT screening programs in Europe for high‐risk individuals. Once implemented, continuous monitoring of participant eligibility, lung cancer detection rate, false‐positive/negative rates, LCS interval, adherence and referral rate, and CT radiation exposure will be required to ensure efficacy.

## Conflict of interest

The authors declare that they have no conflicts of interest.

## Author contributions

Harriet L. Lancaster: conceptualization; writing ‐original draft; writing – review and editing. Marjolein A. Heuvelmans: conceptualization; supervision; writing ‐original draft; writing – review and editing. Matthijs Oudkerk: conceptualization; supervision; writing – original draft; writing – review and editing.
